# Chicago Medical Society

**Published:** 1884-01

**Authors:** 


### Society Reports.

---

### ARTICLE IX.

CHICAGO MEDICAL SOCIETY.

A very large attendance of members of the Chicago Medical Society were present at the regular semi-monthly meeting, held on the evening of November 19, for the transaction of considerable important business and hearing a number of scientific essays. The President, Dr. D. W. Graham, presided. In the absence of the Secretary, Dr. L. H. Montgomery, upon request of the Society, Dr. James E. Henderson acted as Secretary *pro tem.*, and subjoined is the report of the proceedings, considerably abbreviated from his stenographic report:

Drs. Robert H. Babcock and W. L. Copeland, upon recommendation of the Board of Censors, were unanimously elected to membership.

Prof. E. C. Dudley, of the Chicago Medical College, read a paper on "The Immediate Operation for Laceration of the Perinæum." He advocated the operation immediately, or as soon after the confinement as possible, and discussed the subject in all its bearings. But as we expect to have the paper entire for publication in a subsequent number of the JOURNAL, we omit what would be an imperfect synopsis here. The reading of the paper elicited the following interesting discussion:

Dr. George H. Randall was particularly gratified that the matter of faulty and incomplete union of these cases had been brought so prominently before the Society. He himself had thought of the necessity for something more than we have gained by the immediate operation; he desired the author to explain more fully the frequency of the transverse rupture.

Dr. M. H. Thompson thought possibly it made no particular difference whether the operation was deferred for two days after labor or longer. My experience shows that in a much longer time after the operation for laceration than is recommended in the paper, I have placed the knees together, ordered the nurse to keep the parts clean with a douche of carbolized water, and taken all the necessary antiseptic precautions, and I have had perfect union to follow. I have again known cases in which union was obtained, but it occurred irregularly, where perhaps the edge of the mucous membrane would be attached to a quantity of muscular tissue, but the union was firm. I think the patient can be placed in different positions so long as it is done with gentleness, but I do not understand why it is so essential to keep them on the back as stated in the paper.

Dr. R. H. Engert—If the laceration is found to be very deep, if it involves the rectum, I do not think a patient should be operated on under two months, and I will ask the author of the paper if he would operate, under any circumstances, immediately after confinement, no matter how deep the rupture? I lately had a case where there was a laceration in three different places, sideways up into the vagina, about an inch into the rectum, and in the perinæum proper toward the right side, and occurred to a woman with the tenth child. In this case I do not think it would have been practicable to perform an operation right after her confinement, as the parts were so much bruised that it would have endangered success in operating. Three months after the labor I performed an operation with perfectly good results, and which have been maintained up to the present time.

Dr. E. Ingals said: It is perhaps presumptuous for me—a general practitioner—to engage in this discussion, for what can I hope to add to that which has already been said, on a subject that has been so fully treated by those who make a special study of gynæcology. Yet I think there are some useful practices relating to the management of perineal laceration that have not been alluded to. I will say in advance that I am conservative in my views, both in the profession and out of it. I do not easily relinquish old practices. I think the profession is inclined to trust too much to things because they are novel, and about which we hope more than we know; and to reject measures whose usefulness has been demonstrated by long experience. The past history of our profession is full of treasures that have been discarded and that lie neglected all along its pathway. For many years I have attended such a number of obstetric calls as naturally fall to the lot of a general practitioner. Of course, I have seen a good number of perineal lacerations. I will tell you what I have not done for them, and the results. I have not bound the limbs together, I have not required the patient to lie on her side, but have allowed her to take the position that she found to be the most comfortable. I seldom tell her that anything is wrong. If the laceration is extensive I may say to the nurse, "The skin is a little torn; see that the parts are kept scrupulously clean." No woman that I ever attended has had the primary operation performed, nor do I know of one who has had it done secondarily. I do not believe that any one of them would have been benefited by either operation. I can recall but a single case that required anything except cleanliness. This was a primipara, in whose case a severe labor and instrumental delivery resulted in extensive laceration. The wound did not heal kindly, and I stimulated the granulations by a few applications of nitrate of silver. She has since borne a number of children in rapid succession, and has had excellent health. I have cases of laceration that have remained under my observation twenty-five years, and they have experienced no trouble from it. I have had no case that I think would not have been injured by the primary operation. This operation must give the patient some shock and inconvenience, and it may increase her septic dangers, and all this when she is just emerging from the pangs and perils of labor. One-half of the cases of confinement in Chicago are attended by midwives, and I do not suppose any of them have this operation performed. I have consulted a number of the older practitioners of this city, and they all manage their cases just as I do mine, and are rewarded with like results. We should not forget how well the lacerated perinæum will do when left to the reparative processes of nature alone. I am embarrassed in not being able to assent to the teachings on this subject of eminent gynæcologists, but I should feel that I had done less than my duty, if I neglected to say a word in behalf of the poor women who bear our children.

Dr. J. H. Etheridge at this point asked the doctor—you say you never saw a case benefited by the operation? Answered—No case that I have ever attended in my own practice; but if the laceration is very extreme, and consequent prolapsus has occurred, then no doubt the operation would be beneficial. No case in my own practice, primarily, I am sure—and so far as I know, secondarily—has been benefited by the operation.

Dr. L. A. Harcourt—My own experience has been rather limited, but for seven or eight years, I think, I never saw a case of rupture of the perinæum. I said to some of my neighbors, physicians, I have never in my practice had a case of this kind. I was cautioned to examine my patients after confinement. Heeding this caution, and examining them carefully, I found quite a number, and for the past three years I have operated in every case where I was permitted; what might be called the immediate operation. In some cases I delayed twenty hours; not longer, and in every case union by first intention was obtained. The gentleman preceding me spoke of the fear of alarming patients by telling them a serious accident has occurred. It is not my practice to tell a patient in a way that would alarm her. I have practiced the method spoken of in the paper, of paring the edges of the laceration, and I always use the deep sutures, passing them completely beneath the laceration; this, with good care, and the patients have all recovered. I have had only one case of "central laceration," which occurred eight years ago. The labor was quite easy, the child was small, and I was astonished to find a laceration. On examination next morning, I found a small perforation, closer to the vaginal outlet than to the rectum. In that case I did nothing but use a mild application of nitrate of silver, so as to favor healing of the wound, and it proved a success. I think the danger of septic poisoning is overestimated. A year ago last March I was suffering from inflammation of the eyes, and was called upon to attend a woman in confinement. The child was very large. The head was born without any rupture to the perinæum; the cord was twisted around the child's neck, and I made an attempt to draw the cord down, so as to save the child from strangulation. Pains came on suddenly, the body was forcibly expelled, and tore the perinæum through entirely, the anterior half for three-quarters of an inch.

I was in no condition to perform an operation, and summoned a brother practitioner, who kindly consented to do so for me, if the patient did not object. Her friends, however, refused; she neglected every precaution in regard to cleanliness, and yet the woman made a good recovery. She came to me afterward and complained that she could not retain fæcal matter, but appeared to be in good health. Now, that was a most favorable case for blood-poisoning. All the surroundings were most favorable for it; no measures were taken to keep the parts clean. I am, therefore, in favor of the immediate operation in almost every case, for my own all resulted favorably.

Dr. T. P. Seely—My experience is, in succeeding to produce good results, simply by placing the patient on the side, fixing the limbs together, closing the parts and keeping them in apposition for a few days, and observing strict antiseptic precautions; and I would like to ask the author, while I am on the floor, why he places the patient on the "dorsal decubitus" during the operation, as I do not understand how he fixes the patient afterward.

Dr. Dudley, in closing the interesting discussion, of which the foregoing is a resume, said, relative to the frequency of the transverse rupture of which one member asked, that he was unable to answer positively. Within the past year, however, he has seen at the Mercy Hospital four or five cases of this kind; also, he had met with two cases in private practice. I am, therefore, inclined to think this form of rupture is quite common, Thinks incomplete central rupture of the perinæum is the form which most often occurs; have noticed cases in which central rupture had occurred, and in which the posterior commissure had not been ruptured at all. Under these circumstances the rupture is always overlooked, unless very careful examination is made with one finger in the vagina and one in the rectum. If Emmett's idea is not very much the best, then the basis of it may rest in the fact that in incomplete rupture the transverse variety is the most ordinary form. I do not know whether this is the case, but when that form of rupture occurs, the perinæum divides in front so that the parts go to either side, and the shape of the wound is like the letter T, as was stated in the paper. Moreover, I do not mean to say positively that after contraction of the cicatrix, that it will be a cause of a smaller perinæum, but it may be a possible cause, and as one of the speakers asked, if I would operate within two days after laceration occurred? I can reply by stating, Yes, or sooner; and if not called sooner, I should operate on the strength of my experience. I prefer the dorsal position, with the thighs flexed and a small roll placed under the knees. This is the best position for the patient. There is no objection to placing a patient on the side if she again will assume the dorsal position; and the patient generally finds the latter the easiest. I always place a bandage around the knees and legs to keep them constantly in tbe same position. My experience in the primary operation for complete rupture through the sphincter ani muscle is confined to one case, in which union occurred. Apparently, the union of the perinæum was pretty good, but not as good as if a better operation had been performed, there being a slight recto-vaginal fistula left. The patient has had several children since. I think I will make this statement in general, that I would operate in every case of ruptured perinæum, especially if the rupture involved the deeper structures of the vaginal portion, but where rupture occurs through the sphincter ani muscle, in a majority of cases union does not take place, although cases of this kind are on record in which union of this sphincter has occurred. There are cases in which the internal sphincter muscle has been equal to the retentive needs of the patient. Regarding the needle to be used, the one used by Thomas, Emmett and others, is a straight one. The reasons for preferring it are the following: A straight needle can always be introduced in such a way as to know where the point is if you know its length. The curved needle is much larger, and makes a larger incised wound after every suture. The straight needle is perfectly round, and makes a punctured wound, and, again, *per se*, there is an unnecessary amount of damage done with a curved needle. This central transverse rupture is one in which it is difficult to see how the parts can be kept in contact by tying the knees together. The tissues retract afterward, and tying the knees together would have no influence at all unless with the vaginal portion of the wound; neither would sutures passed through the cutaneous surface of the wound—only sutures passed in the axis of the vaginal outlet could, under these circumstances, bring that torn tissue down to the place from which it was torn.

A paper on Glycosuria was next read by Dr. Oscar C. DeWolf, (see this paper) published in the *Journal of the American Medical Association*, Nov. 24, 1883. Discussion upon this very important subject was deferred until the next meeting.

Dr. F. E. Wadhams was to have presented a paper on "Nerve-Stretching" for the relief of an obstinate case of sciatica, but the late hour precluded its being read.

The resolutions presented at the last meeting regarding "The Nucleus of a Medical Library" were taken from the table, and Dr. Edmund Andrews said, "the formation of a medical department in the public library, accessible to every family physician in the city, is a greater public necessity than the duplication of a vast number of novels." The result of the conference with the public library board was that the books would have to be presented and not loaned to the library. This Society would incur no expense in taking care of them. He moved, therefore, the passage of the resolutions reported at the last meeting, and that $500 be appropriated for the purchase of books and journals, which was easily carried; also, that a committee of three on Library be now appointed by the chair, resulting in Drs. E. Andrews, F. C. Hotz and O. C. DeWolf forming the committee for three years, two years, and one year each, in the order their names appear. Dr. J. G. Kiernan wished to have a committee on Medical Legislation appointed, as many of the laws relating to our profession in this State are in a very unsatisfactory condition. Particular reference was made to the confidential relations between physician and patient, and the former's appearance on the witness stand, as any member of this Society may be compelled to reveal the secrets of a patient. For this and similar reasons he offered the above suggestion in the form of a motion, to determine what measures are needed to remedy existing laws on this subject. The motion was unanimously carried. The chair announced that he would appoint the committee at the next meeting.

Another motion made and seconded that we do now adjourn, was also nnanimously tarried. L. H. M.

---

PRIAPISMUS CAUSED BY LEUKEMIA.

The patient, a Hungarian, 35 years of age, suffered from intermittent fever, and a bronchial catarrh for six months, and at this time there were daily erections lasting two or three hours, but during the last two weeks the erections were perpetual. The glans penis takes no part in the erection, and the urethra is soft and sometimes compressed to such a degree that catheterization is necessary. The spleen is enormously enlarged and the white blood corpuscles are multiplied as 1 to 1. The connex between the two conditions is not known.—*Pest. Med. Chir. Pr.*

COFFEE IN STRANGULATED HERNIA.

Dr. Antonio P. Sarra was called to attend a man 63 years old, affected with a right crural hernia. He found the patient in a cold state, without an appreciable radial pulse, with a pinched face, and frequent efforts at vomiting. He called to mind a case successfully treated by Dr. Foussagrives with coffee, and published in 1880, and therefore ordered an infusion of the berry to be taken internally, and also to be applied externally. He left the patient, and notified the family that if a prompt improvement did not ensue death would be inevitable. The next morning he found the patient in excellent condition. On inquiry, it was learned that after taking the coffee he felt immediately refreshed and stronger, passed a great deal of gas by the stomach and by the rectum, felt a "roaring" in his intestines, and having place his hands on the tumor, the hernia was immediately reduced.—*Siglo Medico*, *Lyon Medical.*

